# The Role of the Endothelium in HPS Pathogenesis and Potential Therapeutic Approaches

**DOI:** 10.1155/2012/467059

**Published:** 2012-06-28

**Authors:** Irina Gavrilovskaya, Elena Gorbunova, Valery Matthys, Nadine Dalrymple, Erich Mackow

**Affiliations:** Department of Molecular Genetics and Microbiology, Stony Brook University, Stony Brook, NY 11794-5222, USA

## Abstract

American hantaviruses cause a highly lethal acute pulmonary edema termed hantavirus pulmonary syndrome (HPS). Hantaviruses nonlytically infect endothelial cells and cause dramatic changes in barrier functions of the endothelium without disrupting the endothelium. Instead hantaviruses cause changes in the function of infected endothelial cells that normally regulate fluid barrier functions of capillaries. The endothelium of arteries, veins, and lymphatic vessels is unique and central to the function of vast pulmonary capillary beds, which regulate pulmonary fluid accumulation. The endothelium maintains vascular barrier functions through a complex series of redundant receptors and signaling pathways that serve to both permit fluid and immune cell efflux into tissues and restrict tissue edema. Infection of the endothelium provides several mechanisms for hantaviruses to alter capillary permeability but also defines potential therapeutic targets for regulating acute pulmonary edema and HPS disease. Here we discuss interactions of HPS causing hantaviruses with the endothelium, potential endothelial cell-directed permeability mechanisms, and therapeutic targeting of the endothelium as a means of reducing the severity of HPS disease.

## 1. Introduction

Hantaviruses predominantly infect microvascular endothelial cells (ECs), which line vessels and nonlytically cause two vascular diseases: hemorrhagic fever with renal syndrome (HFRS) and hantavirus pulmonary syndrome (HPS) [1–14]. The mechanisms by which hantaviruses cause capillary leak syndromes and disrupt fluid barrier integrity of the endothelium are beginning to be disclosed and appear to involve dysregulating EC functions that normally limit fluid leakage from the vasculature [6, 15–21]. 

Capillaries, veins, and lymphatic vessels are lined by a single layer of ECs which collectively form one of the largest tissues of the body [22, 23]. The endothelium forms a primary fluid barrier within vessels but serves as more than just a conduit for blood to reach and return from organs [22, 24]. The endothelium selectively restricts blood and plasma from entering tissues, regulates immune cell infiltration, and responds to damage by limiting leakage, repairing vessels, and directing angiogenesis [22]. Consequently, capillary barrier integrity is redundantly regulated by an array of EC-specific effectors that coordinately balance vascular fluid containment with tissue-specific needs and respond to a host of systemic and locally generated factors that alter inter-endothelial cell adherence junctions [22, 25–37]. ECs respond to activated platelets and immune cells, clotting cascades, chemokines and cytokines, growth factors, nitric oxide, and hypoxic conditions [22, 27, 29, 38–41]. However, ECs also secrete cytokines, complement and growth factors that positively or negatively impact the adherence and activation of platelets and immune cells, regulate responses to hypoxia, and diminish or enhance extravasation of fluid into tissues [22, 24, 26, 27, 30, 40–45]. Each of these EC responses is controlled by a diverse mesh of intertwined sensors and signals aimed at returning the endothelium to a resting state, countering permeabilizing effectors, repairing vessel damage, and restoring fluid and oxygenation levels within tissues [22, 25, 39, 41, 46–51]. 

The endothelium of capillaries, veins, and lymphatic vessels are unique and central to discrete functions of vast renal and pulmonary capillary beds [42, 52–54]. Nonlytic viral infection of ECs may disengage one or more fluid barrier regulatory mechanisms, thereby increasing vascular leakage or fluid clearance and as a consequence result in tissue edema [55–60]. Although the edematous accumulation of interstitial fluids can result from increased endothelial permeability, a decrease in lymphatic vessel clearance of tissue fluids is also a cause of edema and regulated by unique lymphatic endothelial cells (LECs) [42, 53, 54, 61]. Vascular permeability induced by nonlytic viruses is likely to be multifactorial in nature, resulting from virally altered EC responses, immune cell and platelet functions, hypoxia, or a collaboration of dysregulated interactions that impact normal fluid barrier function [15–18, 20, 27, 62–64]. Failure of the endothelium to regulate fluid accumulation in tissues has pathologic consequences and during HPS results in localized vascular permeability and acute pulmonary edema that contribute to cardiopulmonary insufficiency [4–6, 9]. Here we focus on the mechanisms by which HPS causing hantavirus infection of ECs induces vascular permeability and acute edema and discuss potential therapeutic mechanisms that may stabilize the endothelium.

## 2. Hantavirus Infection and HPS Disease

Hantaviruses are enveloped, tripartite, negative-sense RNA viruses and form their own genus within the *Bunyaviridae* family [14, 65]. Hantaviruses are the only members of the *Bunyaviridae* that are transmitted to humans by mammalian hosts, and hantaviruses contain highly divergent RNA and protein sequences, which are likely the result of coadaptation with their hosts [13, 14, 66–68]. Single genes have been exchanged between closely related HPS causing hantaviruses [69]; however, gene reassortment has not permitted the discovery of pathogenic determinants and reverse genetics approaches have thus far proven elusive. 

The hantavirus genome consists of three segments denoted S, M, and L based on the length of their RNA segments, respectively [14]. The L segment encodes the 250 kDa RNA-dependent RNA polymerase [14, 67, 70]. The S segment encodes a 48 kDa nucleocapsid (N) protein which is the most abundant hantavirus antigen present in infected cells [14, 70, 71]. The M segment encodes two viral surface glycoproteins Gn (64 kDa) and Gc (54 kDa) that are cotranslationally cleaved and targeted to the ER/cis-Golgi [14, 72]. Hantaviruses bud internally into the lumen of the cis-Golgi and exit cells via a secretory mechanism consistent with aberrant vesicular trafficking [14]. Hantaviruses are both released from ECs and remain cell associated through interactions with cell surface receptors [15, 62, 73]. GnGc heterodimers on the virion surface are presumed to bind cellular receptors and mediate viral entry into cells [14, 15, 20, 72–79].

Hantaviruses are one of only a few viruses that primarily infect the EC lining of the vasculature [8, 9, 12, 80, 81]. Hantaviruses replicate to low titers, with initial viral progeny emerging from infected ECs 18–24 hours postinfection (hpi), and ~5 × 10^6^ maximal titers days after infection [14]. Infection of ECs is nonlytic and the permeability of infected EC monolayers is not increased by infection alone [14, 16, 82]. Prototypic HFRS (Hantaan virus-HTNV), HPS (SNV, ANDV, NY-1V) [3, 5, 10, 83, 84], and nonpathogenic (Tula virus-TULV and Prospect Hill virus-PHV) [85–87] hantaviruses all infect human ECs regardless of their ability to cause disease, suggesting that EC entry alone is not a cause of pathogenesis [12, 16, 81]. At least two requirements for hantaviruses to be pathogenic have been determined thus far, the ability of hantaviruses to regulate early interferon responses and the use of specific integrins by pathogenic (ANDV, SNV, NY-1V, PUUV, SEOV, HTNV) but not nonpathogenic (PHV, TULV) hantaviruses [20, 76, 77, 79, 88–91]. 

### 2.1. Endothelial Cell Attachment and Entry

The cellular entry of pathogenic hantaviruses is dependent on the presence of *α*_v_ *β*_3_ integrins on human ECs, while nonpathogenic hantaviruses PHV and TULV use *α*_5_ *β*_1_ integrins [76, 77]. Cells expressing recombinant *α*_v_ *β*_3_ or *α*_IIb_ *β*_3_ facilitate infection by SNV, NY-1V, ANDV, and HTNV, and infection is blocked by antibodies to *β*_3_ integrins and by the *β*_3_ integrin ligand vitronectin [62, 76]. NY-1V and ANDV bind *β*_3_ integrins in an RGD-independent fashion via interactions with plexin-semaphorin-integrin (PSI) domains present at the apex of bent, inactive, *α*_v_ *β*_3_ integrin conformers [20, 79, 92–95]. Hantavirus binding to  *β*_3_ maintains the integrin in a resting state and inhibits EC migration on the high-affinity *α*_v_ *β*_3_ integrin ligand vitronectin [20, 62]. Infected ECs contain cell associated hantaviruses on their surface at late times post infection [15, 62, 73]. Cell associated pathogenic hantaviruses further direct the binding of quiescent platelets to the EC surface [15]. This interaction may mask hantavirus infected cells or contribute to thrombocytopenia, which is a prominent feature of hantavirus patients. Curiously, *α*_v_ *β*_3_ integrins present on ECs normally regulate vascular permeability, and inhibiting *β*_3_ integrin functions alone causes vascular permeability disorders [41, 95–100]. Links between hantavirus dysregulation of *α*_v_ *β*_3_ functions and their role in EC permeability are discussed in detail below. 

### 2.2. HPS Disease

At least 17 hantaviruses cause HPS, also termed hantavirus cardiopulmonary syndrome (HCPS), with prototype HPS viruses, Sin Nombre (SNV) in North America and Andes (ANDV) in South America [4–9, 101–104]. ANDV, SNV, and many closely related hantaviruses cause HPS resulting in acute pulmonary edema, cardiopulmonary insufficiency, and ~35–40% mortality rates [4–9, 13, 101–103, 105–109]. One to two weeks after infection, there is a rapid onset of pulmonary edema and hypoxia that occurs 6–12 hours after cough and rapidly progresses in severity [4–6]. Bilateral pulmonary infiltrates may be interstitial or alveolar with liters of pleural effusions observed during SNV infection [4–6, 8, 9].

Hantavirus antigen is found predominately in vast pulmonary capillary EC beds but is present in ECs within lymph nodes and throughout the body [8, 9, 80]. However, cytopathic effects are not evident following hantavirus infection of ECs *in vitro* or *in vivo* [9, 16, 82]. Histologically, the heart, kidneys, brain, and adrenals are grossly normal with pulmonary alveoli filled with acellular proteinaceous fluid, yet the alveolar epithelium remains intact [4–6, 8, 9]. The most striking HPS findings are edematous lungs with up to 8 liters of pleural edema [5, 6, 8, 9]. Pulmonary edema fluid contains few leukocytes, is largely serous in nature, and is consistent with the nearly complete loss of an alveolar capillary fluid barrier [4–6, 9]. The lack of disrupted endothelium during HPS is similar to edematous pulmonary responses observed in patients with high altitude-induced pulmonary edema [40, 60, 110–112]. The rapid onset of edematous symptoms during hantavirus infection [6] further suggests the importance of targeting vessel stability in regulating highly lethal hantavirus disease.

## 3. Vascular and Lymphatic Endothelium:**** Control of Vascular Fluid Barrier Functions

The endothelium lines a series of discrete vessel types that conduct fluids to and from tissues, directs the transfer of nutrients, wastes and oxygen and coordinates tissue responses to changing conditions and pathogens [22, 24, 27, 54, 59, 113–119]. Vascular ECs serve mainly as a conduit in the lining of high pressure arteries but take on a variety of fluid and cellular barrier functions in low pressure veins and capillaries that innervate organs and tissues [54]. Lymphatic vessels have a primary role in draining fluid, proteins, and immune cells from tissues and returning these components to the venous circulation [42, 52–54, 114]. Depending on their location, lymphatic vessels serve discrete fluid barrier and regulatory functions, keeping pulmonary alveolar spaces dry and clearing fluid influx from the lungs [54, 61, 120]. These diverse EC settings require discrete EC functions to effect exchange within large capillary beds of the kidney, liver, and lung [27, 54]. 

The EC lining is responsible for controlling vessel damage through a complex mechanism of negative regulation, rapid response and proliferation [22, 24, 40, 53, 120, 121]. Unless activated, the endothelium normally prevents immune cells and platelets from adhering to its surface [22, 122]. Endothelial quiescence is maintained by several mediators, while vascular injury activates clotting factors, platelets, and ECs resulting in the recruitment of platelets to the endothelium [22]. ECs also have angiogenic roles migrating and proliferating to fill in endothelial cell gaps or to rebuild damaged vessels [29, 123]. EC migration and vessel remodeling requires changes in cell adherence within the endothelium, and carefully orchestrated receptor signaling responses are required to accomplish this without causing edema. 

The endothelial fluid barrier is primarily derived from unique adherens junctions (AJs) composed of an EC-specific vascular-endothelial cadherin (VE-cadherin) [25, 30, 45, 96, 117, 119, 124]. EC barrier functions are increased by the presence of cell surface VE-cadherin and reduced by the dissociation and internalization of VE-cadherin [25, 30, 117]. Phosphorylated VE-cadherin is internalized by its interaction with intracellular actin complexes and this process is regulated by a variety of cellular receptors and intracellular signaling pathways [25, 124, 125]. VE-cadherin phosphorylation is downregulated by an EC-specific phosphatase (VE-phosphatase) [124, 125] and several pathways that either directly or indirectly induce AJ assembly and EC integrity by returning VE-cadherin to an unphosphorylated resting state [25, 117, 125]. Chemokines, cytokines, and growth factors indirectly act on EC adherens junctions to increase vascular permeability and thus have the potential to contribute to pathogenic vascular leakage [27, 29, 96]. 

## 4. Unique Receptors Regulate EC Permeability

The endothelium contains many unique receptors that regulate AJ assembly and positively or negatively impact AJ stability and vascular integrity [33, 96, 126–129]. Vascular endothelial growth factor (VEGF) binds to EC-specific VEGFR2 receptors and activates a Src-Rac-Pak-VE-cadherin pathway resulting in AJ disassembly and vascular permeability [25, 30, 117]. Specialized ECs contain unique VEGFR1/2/3 that respond to novel forms of VEGF (VEGF A-E) and control AJ disassembly [61, 96, 130]. LECs uniquely express VEGFR3 on their surfaces and respond to VEGF-C/D but also coexpress VEGF-A responsive VEGFR2 receptors and are further regulated by the formation of VEGFR 2/3 heterodimers [39, 42, 53, 131]. 

VEGF was originally discovered as a potent vascular permeability factor that induces acute edema [29, 132]. VEGF reportedly acts within 0.5 mm of its release [133], and circulating soluble VEGFRs prevent VEGF from systemically permeabilizing vessels [39, 132]. VEGF is induced by hypoxia, and reduced oxygen levels at high altitudes cause high-altitude-induced pulmonary edema (HAPE) [35, 40, 113]. This results from activating the hypoxia-induced transcription factor-1*α* (HIF-1*α*), which senses oxygen levels and transcriptionally induces VEGF [59, 128, 134, 135]. VEGF further upregulates HIF-1*α*, forming an autocrine loop, which amplifies hypoxia-mediated VEGF responses and causes HAPE [59, 113, 136, 137]. Although this response fosters increased gas exchange, in continued low-oxygen environments these cellular responses, instead cause pulmonary edema and in HAPE, respiratory distress [40, 110, 113, 128]. 

As part of the normal process of vascular repair and angiogenesis, ECs migrate in response to VEGF-A stimulation in the presence of extracellular matrix [41, 95, 138]. Permeabilizing VEGFR2 responses are normally controlled by specific cell surface integrins that modulate VEGFR2 complex formation, signaling and permeability responses [96, 97, 116, 139, 140]. Ectodomains of *α*_v_ *β*_3_ and VEGFR2 form complexes that direct EC migration, a process that requires AJ disassembly, yet need to limit VEGF-A-induced permeability [96, 139]. Knocking out *β*_3_ integrins or antagonizing *α*_v_ *β*_3_  results in enhanced VEGF-A directed permeability of capillaries *in vivo* and *in vitro* [97, 141, 142]. *α*_v_ *β*_3_  antagonists are reported to promote the rapid recycling of internalized VEGFR2 to the cell surface, amplifying EC responses to VEGF [96, 97, 143]. 

*β*_3_ integrin functions are further regulated by interactions with cell surface syndecan-1 and additional interactions of neuropilin-1 (Nrp-1) with VEGFR2 [127, 142, 144–147]. Nrp-1 is a VEGF-A coreceptor that forms an ectodomain complex with VEGFR2 that regulates the permeabilizing effects of VEGF [127, 142, 144, 145], and Nrp-1 binding to VEGFR2 is further regulated by the binding of semaphorin3A (sema3A) [145–147]. Endothelial roundabout receptors, Robo1 and Robo4, also impact VEGFR2-directed permeability through discrete signaling pathways [36, 48, 148–150]. Slit-2 binding to Robo1 and Robo4, respectively, have positive or negative effects on VEGF-A directed EC permeability [151, 152]. However, Robo 1/4 are differentially expressed in discrete EC beds suggesting the localized permeability effects of slit-2 [148, 152]. These findings indicate that many EC responses control capillary leakage through interconnected mechanisms and suggest that altering any number of orchestrated EC barrier functions can result in edema. 

## 5. Hantavirus-Endothelial Cell Interactions

### 5.1. Hantavirus Binding to Inactive *α*_*v*_ *β*_3_  Integrins Regulates EC Functions and Permeability

Pathogenic hantaviruses bind to inactive, basal conformations of *α*_v_ *β*_3_  integrin receptors on ECs, while nonpathogenic hantaviruses interact with discrete integrins [76, 77, 79]. Receptor binding directs viral entry, but at late times postinfection cell associated hantaviruses also negatively impact  *α*_v_ *β*_3_  integrin functions [15–18, 20, 62]. Days after infection, cell-associated pathogenic hantaviruses block  *α*_v_ *β*_3_  integrin directed EC migration and direct the binding of quiescent platelets to the EC surface [15, 62]. Similar to antagonizing or knocking out  *α*_v_ *β*_3_  integrins [96, 97], pathogenic hantavirus infection of human ECs sensitizes monolayers to the permeabilizing effects of VEGF [16, 17]. SNV-, ANDV-, and HTNV- infected ECs, but not nonpathogenic PHV or TULV infected ECs, are hyperresponsive to the permeabilizing effects of VEGF [16], and VEGFR2 is hyperphosphorylated following pathogenic hantavirus infection [15, 17, 18]. Additionally, enhanced permeability of infected ECs only occurs days after infection when cell-associated hantaviruses coat the cell surface and inactivate  *α*_v_ *β*_3_  integrins [15–17, 20, 73]. These findings, in the context of hypoxic HPS patients, suggest that hantavirus binding to inactive *α*_v_ *β*_3_  integrins contributes to capillary permeability in HPS. These results further suggest a mechanism for hantavirus-enhanced EC permeability that stems from disrupting normal *α*_v_ *β*_3_-VEGFR2 interactions and enhanced VEGFR2-Src-VE-cadherin signaling responses that dissociate VE-cadherin from AJs [15–17, 20, 25, 96].

One paper suggests that ANDV-infected ECs transiently induce VEGF secretion, VE-cadherin degradation, and increased EC monolayer permeability [21]. However, several studies indicate that monolayers of hantavirus-infected ECs are not permeabilized by infection alone [16, 17, 82] and instead indicate that pathogenic hantavirus infected ECs are hyperpermeabilized by VEGF [16]. Collectively, these findings demonstrate that cell surface hantaviruses alter normal EC functions that control VEGF-directed vascular permeability [15–18, 62, 153].

### 5.2. Potential Role of LECs in Hantavirus Edema

Pulmonary lymphatic vessels are responsible for clearing fluid from alveoli and providing a semidry state that permits gas exchange [52, 54]. Failure of lymphatic vessels to clear fluids results in lymphedema and suggests an additional mechanism for hantavirus-infected LECs to contribute to acute pulmonary edema during HPS [42, 53, 54, 154]. Analysis of pathology samples from HPS patients indicates that hantavirus antigen is present in LECs of patient lymph nodes [8, 9, 80]. Although less is known about LECs, as described above, LECs express unique cell surface receptors and their integrity is regulated by both VEGF-A and VEGF-C [42, 53, 54, 61]. Interestingly, LEC VEGFR3 receptors respond to VEGF-C and are associated with reduced tissue edema [42, 61], while inhibiting VEGFR3 signaling results in lymphedema [42, 131]. Although a recent publication indicates that ANDV infects LECs and alters LEC barrier functions [155], the role of lymphatic vessels and LEC responses remains to be investigated in HPS patients.

### 5.3. Hantavirus-Endothelial Edemagenic Mechanisms

Prominent pulmonary and renal dysfunction are components of both HPS and HFRS diseases and likely stem from hantavirus infection of ECs, which line vast alveolar and renal capillary beds [4–6, 8, 9, 156, 157]. HPS patients are often young adults that arrive at hospitals in acute respiratory distress [4]. Acute pulmonary edema is a hallmark of HPS, with bilateral fluid infiltrates accumulating at up to a liter per hour resulting in pulmonary insufficiency and patient hypoxia during a critical phase of the disease [4, 6, 8, 9]. The cause of acute edema following hantavirus infection is likely to be multifactorial [6, 15–18, 64, 153, 155, 158] but revolves around the ability of the hantaviruses to infect ECs within alveolar capillary beds that normally regulate edema and gas exchange within the lung. 

Clues to the mechanism of hantavirus-induced edema come from disparate findings on the role of hypoxia in acute pulmonary edema and the role of *α*_v_ *β*_3_  and VEGFR2 EC responses, which are uniquely altered by pathogenic hantaviruses [6, 15, 16, 20, 155]. Hypoxia is a prominent component of HPS patients and directs VEGF secretion from endothelial, epithelial, and immune cells [5, 6, 8, 9]. Consistent with the enhanced permeability of hantavirus-infected ECs in response to VEGF [16], HPS may be the result of hypoxia-induced VEGF that leads to acute pulmonary edema and may be exacerbated by reduced lymphatic vessel fluid clearance [155]. In fact, HPS patient VEGF levels were markedly elevated in pulmonary edema fluid and PBMCs in acute early phases of HPS [159]. Although a demonstrated role for hypoxia in hantavirus-induced permeability has yet to be conclusively defined, the ability of extracorporeal membrane oxygenation (ECMO) to reduce HPS patient mortality [4, 6] strongly suggests a role for hypoxia and VEGF in the acute pulmonary edema of HPS patients.

## 6. Animal HPS Model

Only ANDV infection of Syrian hamsters (*Mesocricetus auratus*) serves as a model of hantavirus pathogenesis that mimics human HPS in onset symptoms and lethal acute respiratory disease [19, 160, 161]. Inoculation of Syrian hamsters with ANDV, but not SNV or other HPS causing hantaviruses, induces pathology approximating human disease. ANDV causes a fatal infection of Syrian hamsters with an LD_50_ of 8 plaque-forming units. The disease is characterized by large pleural effusions, congested lungs, and interstitial pneumonitis in the absence of disrupted endothelium [19, 160, 161]. The onset of pulmonary edema coincides with a rapid increase in viremia on day 6, and large inclusion bodies and vacuoles in ultrastructural studies of infected pulmonary ECs [160, 161]. Viral antigen was localized to capillary ECs, alveolar macrophages, and splenic follicular marginal zones populated by dendritic cells. Interestingly, depletion of CD4 and CD8 T-cells had no effect on the onset, course, symptoms, or outcome of ANDV infection and indicates the absence of T-cell responses [19]. Consistent with the potential involvement of *β*_3_ integrins and VEGF in this process, ANDV binds to conserved residues within PSI domains of both human and hamster *β*_3_ integrins [20, 79]. Thus the mechanism of pathogenesis caused by ANDV is consistent with hypoxia-VEGF- directed acute pulmonary edema that occurs in the absence of T-cell-mediated pathology [19]. These findings differ from a report associating T-cell responses with HPS disease, although the same data support a lack of T-cell involvement, since half of HPS patients had no elevated T-cell responses regardless of disease severity [64]. Observed T-cell responses may instead correlate with viral clearance [63, 162]. The mechanism of pathogenesis may be further elucidated by studies in Syrian hamsters and thus provides a model of ANDV pathogenesis that permits the evaluation of therapeutics that target barrier functions of the endothelium.

## 7. Targeted Therapeutic Approaches for**** Stabilizing the Endothelium

Currently, there are no effective therapeutics for hantavirus infections or disease. Antiviral effects of interferon or the nucleoside analog ribavirin are only effective prophylactically or at very early times postinfection [14, 163]. They appear to target early viral replication but neither is effective 1-2 weeks postinfection after the onset of HPS symptoms [4–6, 163]. An alternative approach against viruses with a long disease onset may be to therapeutically target the acute pathologic response instead of viral replication. Since hantaviruses infect and alter fluid barrier functions of the endothelium, targeting EC responses that transiently stabilize the vasculature has the potential to reduce the severity and mortality of HPS [50, 129, 164]. This approach also has the advantage of being implemented at the onset of symptoms where antiviral approaches appear to be ineffective [163]. 

Intracellular signaling pathways coordinately regulate the adherence of ECs to the extracellular matrix, anchor receptors to cytoskeletal elements, and induce growth factor directed migration, proliferation and permeability responses [18, 35, 41, 43, 50, 96, 116, 165, 166]. The complexity of VEGF induced permeability is further demonstrated by the reported ability of rapamycin, an inhibitor of mammalian target of rapamycin (mTOR) signaling responses, to block VEGF-induced microvascular permeability [167–171]. This multifactorial coordination indicates why so many factors are capable of permeabilizing or stabilizing the endothelium and rationalizes their potential roles in pathogen-induced capillary leakage. 

Antibody to VEGFR2 reportedly suppresses VEGF-induced pulmonary edema and suggests the potential of therapeutically antagonizing VEGFR2-Src-VE-cadherin signaling pathways as a means of reducing acute pulmonary edema during HPS [18, 25, 39, 50, 172–176]. Several well-studied VEGFR2 and Src inhibitors are in human clinical trials or are used therapeutically to treat human cancers and have the potential to reduce the severity of viral permeability-based diseases [18, 42, 50, 173, 174, 177–179]. *In vitro*, angiopoietin-1 (Ang-1), sphingosine-1-phosphate (S1P), pazopanib, and dasatinib inhibited EC permeability directed by pathogenic hantaviruses [16, 18]. Ang-1 is an EC-specific growth factor that transdominantly blocks VEGFR2-directed permeability *in vitro* and *in vivo* by binding to Tie-2 receptors [180–183]. S1P is a platelet derived lipid mediator, which enhances vascular barrier functions by binding to Edg-1 receptors on the endothelium [47, 172, 173, 179, 184], while pazopanib and dasatinib are drugs that inhibit VEGFR2-Src signaling [174, 185]. Pazopanib, dasatinib, and the S1P analog FTY720 are already in clinical trials or used clinically for other purposes [34, 186]. Targeting EC responses provides a potential means of stabilizing HPS patient vessels and reducing edema. The use of S1P receptor agonists has also been shown to regulate the pathogenesis of influenza virus infection by acting on ECs and reducing immune cell recruitment and entry into the lung [172]. These findings suggest the targeting of EC functions as a means of increasing capillary barrier functions and regulating immune responses that contribute to viral pathogenesis.

The regulation of additional EC receptors that stabilize interendothelial cell AJs and fluid barrier functions of the endothelium may be considered as therapeutic targets. The Robo4 receptor has been shown to inhibit VEGFR2 responses, stabilize vessels and block vascular permeability [48, 148, 152]. This new potential target is highly expressed by lung microvascular ECs and is currently being evaluated as a therapeutic for a variety of vascular disorders [149, 152]. However, Robo4 directed stability of interendothelial cell junctions may also be applicable to reducing HPS severity. 

Several additional EC receptors that bind to VEGFR2 ectodomains positively or negatively regulate *α*_v_ *β*_3_-VEGFR2 functions and may provide additional therapeutic targets for regulating vascular permeability. Potential responses which need to be investigated as therapeutic targets include: NRP1, Syndecan1 (sdc1), and the insulin-like growth factor1 receptor (IGF1R), which are recruited to *α*_v_ *β*_3_ ectodomain complexes [49, 141, 142, 144, 175, 187, 188]: Surfen, a heparan sulfate containing protein that reportedly blocks EC permeability [189], and Fibulin-5, a matrix protein that reportedly promotes EC adherence by binding *α*_v_ *β*_3_ and is associated with emphysema [190–192]. However, inhibiting *β*_3_ receptors that are present on both platelets and ECs may exacerbate permeability and thus the choice of therapeutic targets is likely to be critical to increasing fluid barrier functions of the endothelium. Targeting the VEGFR2 axis that regulates EC permeability may be a central mechanism for stabilizing the endothelium and reducing the severity of HPS [127, 145, 175, 193]. 

These findings suggest a plethora of targets that may regulate virally induced vascular permeability and which are already clinically approved for other indications. Moreover, targeting these responses may be broadly applicable to reducing the severity of HFRS and a wide range of viral infections that impact the endothelium and cause edematous diseases.

## 8. Future Directions and Conclusions

The endothelium plays a fundamental role in vascular disease, and stabilizing the vasculature needs to be evaluated as a means for reducing the severity and mortality of viral vascular diseases. This is especially important for viral infections that cause disease 1-2 weeks after infection, at time points when antiviral approaches are no longer viable. The ability of hantaviruses to infect LECs and alter normal fluid clearance from tissues needs to be investigated and provides a unique target and mechanism for reducing edema that has yet to be considered in HPS disease. The ability of the endothelium to regulate platelet functions, complement activation, and immune responses should also be considered as central targets for reducing the severity of viral hemorrhagic and edematous diseases. 
